# *Actinomyces Meyeri* Empyema Necessitatis—A Case Report and Review of the Literature

**DOI:** 10.1055/s-0039-1693653

**Published:** 2019-07-16

**Authors:** David B. Ellebrecht, Moritz M.F. Pross, Stefanie Schierholz, Emanuel Palade

**Affiliations:** 1 Department of Surgery, University Medical Center Schleswig-Holstein, Luebeck, Germany; 2 Department of Orthopedics and Trauma Surgery, Robert Bosch Hospital, Stuttgart, Germany

**Keywords:** *Actinomyces meyeri*, pleural empyema, empyema necessitatis, antibiotic treatment

## Abstract

Pleural empyema necessitatis caused by *Actinomyces meyeri* is a rare but severe infection. *A. species* predominantly *A. meyeri* and *A. israelii* represent the second most common pathogen for empyema necessitans after mycobacteria. The incidence is reported in the literature to be 1:300,000. Men are thrice more likely to be affected than women. Pathogenetically, an infection can be triggered by aspiration in immunocompromised individuals which leads to an atelectasis with pneumonitis.

In two cases, a 38-year-old construction worker and a 61-year-old woman with ulcerative breast carcinoma, who presented to the local emergency department with a painful swelling of the left chest, diagnostic workup revealed a pleural empyema necessitatis of the left chest. An antibiotic treatment was initiated with piperacillin/tazobactam and sulbactam/ampicillin, respectively. Temporally vacuum-dressing therapy was initiated after surgical debridement. In the course of the procedure, a reconstruction of tissue damage was feasible. The patients were recovered completely and discharged with an oral antibiotic treatment (amoxicillin) for 6 and 12 months, respectively.

Thoracic actinomycosis is a relatively uncommon and traditionally chronic, indolent infection secondary to pulmonary infection with *A. species* . Surgical treatment is generally reserved for cases failing to resolve with antibiotic therapy. Early diagnosis, prompt debridement, and narrow spectrum β-lactam antibiotics can result in complete resolution of infection and good prognosis.

Pleural empyema is associated with mortality rates up to 20% and surgical treatment is indicated when initial drainage therapy is not sufficient or patients develop increasing signs of infection, for example, septic decompensation under ongoing conservative therapy. 1 Surgical intervention is also the treatment of choice for the rare complication of extended local infection through the chest wall known as empyema necessitatis. 2 On one hand the etiology of empyema and, particularly, the causative organism has almost no impact on the initial performed surgical intervention of infected tissue excision. On the other hand, for the further postoperative treatment, especially type and duration of antibiotics, knowledge of the causal agent is crucial. *Actinomyces meyeri* is a filamentous, gram-positive anaerobic bacterium that can be found physiologically in the normal flora of the respiratory and gastrointestinal tract and is rarely pathological. Orocervicofacial, pulmonary, or abdominopelvic infections are the most common causes. *A. species* , predominantly *A. meyeri* and *A. israelii* , represent the second most common pathogen for empyema necessitans after mycobacteria. 3 In most cases, diagnosis and detection of actinomycosis renders complex due to its rareness and unspecific presentation. 4 We hereby report two cases of empyema necessitatis and chest wall abscess secondary to pulmonary actinomycosis caused by *A. meyeri* and present an overview of diagnostics and therapy of actinomycosis.

## Case 1

A 38-year-old construction worker presented to the local emergency department with a progressive, painful swelling of the left pectoral region with a history of about 4 weeks. Additionally, he complained about productive cough with purulent sputum for 4 days. Dyspnea or angina pectoris were negated. In the medical history, there had not been any travels, contact to animals or tuberculosis patients. He had always been in good health and his medical history showed no diseases apart from a nicotine abuse.

**Fig. 1:**
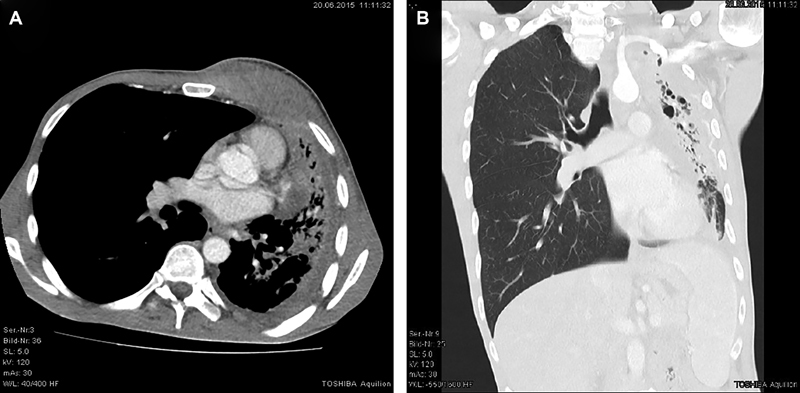
( **A** and **B** ) CT thorax of a 38-year-old patient with retropectoral abscess and empyema necessitation the left side due to extensive pleuropulmonary actinomycosis. The abscess was debrided by surgery several times. Lung resection or decortication has not been performed. After 17 days of vacuum dressing therapy in combination with ampicillin–sulbactam the wound could be closed secondarily. The total duration of the antibiotic therapy was 1 year. CT, computed tomography.

In the emergency room, the cachectic patient was in acute distress. The patient's physical examination revealed a 12 × 10 cm redness and painful abscess of the left pectoral region. The left chest appeared fallen in and smaller than the right side. The chest examination yielded dullness on percussion and an absent breath sound on the left side. The chest X-ray showed an almost complete opacity of the left hemithorax with mediastinal shift to the left side and decreased left intercostal spaces. The right lung was compensatory overinflated. The white blood count showed 16.4 × 10 ^3^ /μL leukocytes and an elevated C-reactive protein (108 mg/L). Liver and kidney function test were within normal limits. A computed tomography (CT) scan revealed an abscess in the left upper lobe surrounded by pneumonic infiltrates and invading the adjacent chest wall and pectoralis muscle ( Fig. 1 ).

An antibiotic treatment with piperacillin 4 g/tazobactam 0.5 g in every 8 hours was initiated and the patient was taken to the operation room. The debridement of the soft tissue and chest wall abscess including the intrapleural necrosis was performed. Keeping *Actinomycosis* as differential diagnosis in mind, no lung or chest wall resection was performed. Wound management consisting of multiple surgical debridement and vacuum dressing system followed. We were able to close the wound 17 days after admission.

Staining and polymerase chain reaction for mycobacterium were negative. The aerobic culture showed no isolated pathogens. However, *A. meyeri* was detected in the anaerobic culture after 48 hours of inoculation. The specimen assessment was not able to reveal any *A. meyeri* .

**Fig. 2:**
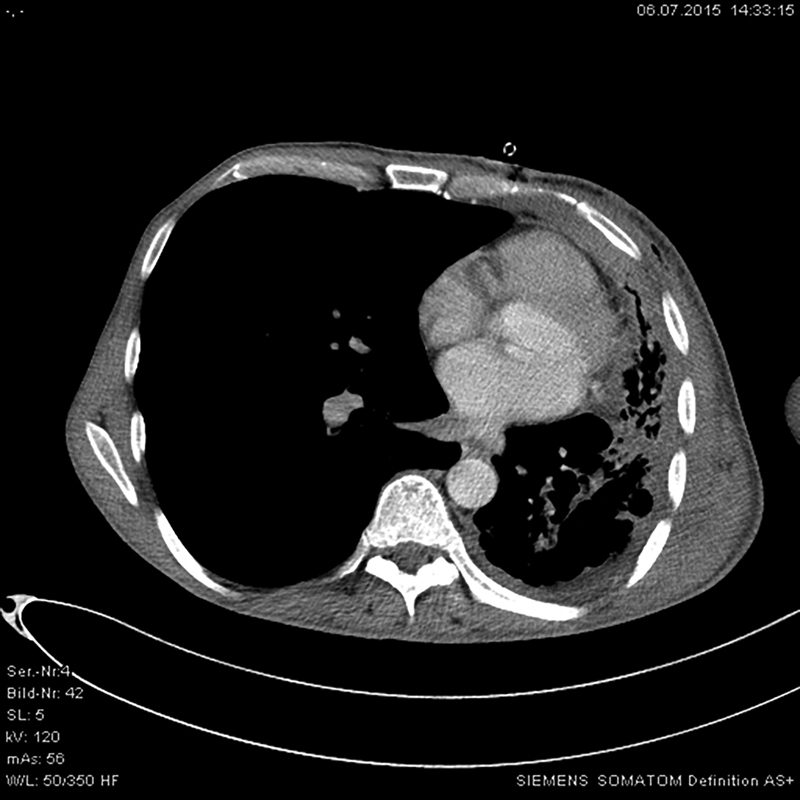
The follow-up examination after 12 months showed recovery of left chest and lung.

The antibiotic treatment, which had been started at the day of admission, was changed to cefuroxime intravenous (IV) after *A. meyeri* had been identified. The patient recovered completely and was discharged 3 weeks after the surgical procedure. Due to the extensive involvement of the left lung, an oral antibiotic treatment (amoxicillin) was continued for 12 months. Treatment duration was decided interdisciplinary. Main criterion was the resolution and stabilization of the inflammatory lesions documented by CT scans. The follow-up examination after 12 months showed recovery of left chest and lung ( Fig. 2 ).

## Case 2

A 61-year-old female presented to an outside hospital with painful swelling of the left chest and left sided ulcerative breast carcinoma with a medical history of coughing about last months. She reported that she had recognized a tumor of the left breast 1 year before admission. But she did not want to clarify her findings.

**Fig. 3:**
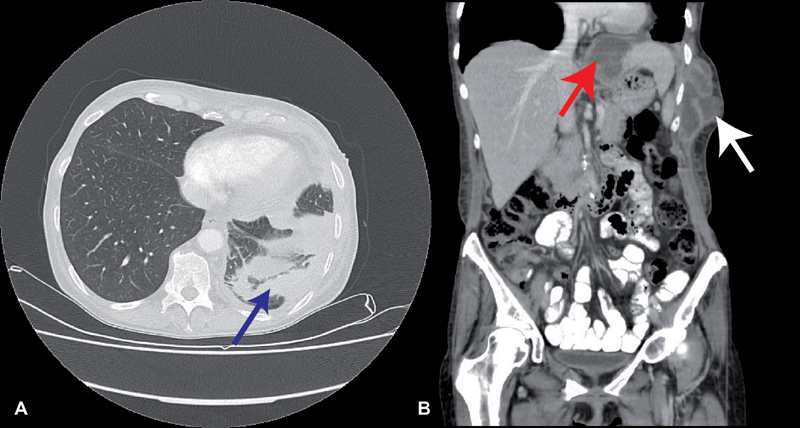
CT-scan of 61-year-old female showed a pleural empyema ( **A** : blue arrow, **B** : white arrow) with expansion per continuitatem to the left chest and left upper abdominal quadrant ( **B** : red arrow). CT, computed tomography.

The white blood count showed 16.4 × 10 ^3^ /μL leukocytes and elevated C-reactive protein of 216.1 mg/L. Initial workup included X-ray imaging of the chest and showed pleural effusion of the left side. Ultrasound of the swelling revealed a large abscess of the left chest with suspicion of communication to the pleural effusion. Following CT showed a pleural empyema with expansion per continuitatem to the left chest and left upper abdominal quadrant ( Fig. 3 ). Puncture of chest abscess formation revealed pus. Microbiological cultures returned positive for *A. meyeri* and *Staphylococcus hominis* . She was started on sulbactam and ampicillin and was urgently transferred to our hospital for further management.

A second CT scan revealed a recession of the pleural empyema and intra-abdominal abscess formation. Therefore, we performed a debridement of the chest abscess formation and excision of the fistula between pleural space and left chest. To close, the chest wound temporarily and to accelerate wound healing, V.A.C. (Vacuum Assisted Closure, V.A.C.ULTA™ Therapy System, KCI Medizinprodukte GmbH) therapy was initiated.

After one cycle of 4 days V.A.C. therapy reconstruction of the skin and soft tissue defects was performed. The patient recovered completely. Workup of the breast carcinoma showed no further metastasis. She was discharged 5 days after wound reconstruction. Additionally, we initiated a gynecological consultation.

**Fig. 4:**
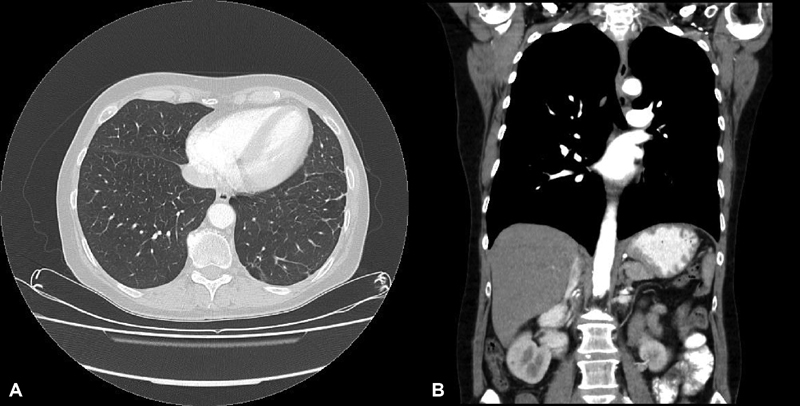
( **A** and **B** ) After debridement of chest abscess formation and excision of the fistula between pleural space and left chest, and intravenous and oral antibiotic treatment for 6 months, follow-up examination showed recovery of chest, lung, and abdomen.

Oral antibiotic treatment was continued for 6 months. Follow-up examination showed recovery of chest, lung and abdomen ( Fig. 4 ).

## Discussion

Despite all efforts and improvements in modern diagnostics, some diseases still remain elusive at the time of initial presentation. Actinomycosis certainly represents one of these entities. The actinomyces bacteria family are filamentous gram-positive, rod shaped anaerobic cells including many species and normally reside as concomitant inhabitants in the human oropharyngeal tract. 5 They can act as opportunistic pathogens and cause infect when the mucosal barrier is disrupted, for example, due to structural diseases or concomitant infections, such as dental infections. *A. species* can migrate into deeper structures and cause infection. 6 In most cases, chronic cervicofascial infection also known as “lumpy jaw” is the result and *A. israelii* is detected. 7 There are few data about the frequency of actinomycosis. An incidence of 1 per 300,000 was estimated in 1970. 8 Probably with improvement in dental hygiene and use of antibiotics the incidence dropped further in developed countries. 9 10 During the review of literature we found six cases of empyema caused by *A. israelii* 11 12 13 14 15 16 and further six cases of empyema due to *A. meyeri* . 6 7 17 18 19 20

Clinical appearance is nonspecific and includes fever, weight loss, productive cough, and chest pain. From a radiological and clinical point of view, it can be confused with neoplasia, for example, lung cancer. The final diagnosis is often made by bronchoscopic or percutaneous needle aspiration. The detection of pathogens is usually better microscopically (direct preparation and gram staining) than culturally. It has to be considered that the *Actinomyces* are very difficult to grow (anaerobic/microaerophilic incubation up to 14 days). Detection and confirmation by Polymerase Chain Reaction (PCR) are reliable but seldom performed, as one has to think about *Actinomyces* . In our cases, culture and PCR on drainage fluid resulted in no evidence of *Actinomyces* but microbiological culture of tissue detected *A. meyeri* . If actinomycosis infections of the pleura and thoracic wall with or without existing cutaneous fistula are suspected, it is advisable to provide tissue samples for the microbiologist and pathologist. PCR (specific nucleic acid detection) is also possible. Radiologic presentation is variable and unspecific so in most cases it does not contribute to the exact diagnosis. 21 Histopathological examination can reveal filamentous structures mimicking fungal growth. Furthermore, sulfur granules and granuloma can be detected, such as in infection with *Nocardia* , coccygea, or aspergilla, which leads to a challenging differentiation. 22

Surgical therapy in complicated thoracic infections, especially in empyema, is the treatment of choice providing rapid focus control and, thus, should be initiated when conservative treatment fails, or infection extends. Additionally, adapted antibiotic therapy is required. 23 For most cases with actinomycosis, due to the high susceptibility of these germs to penicillin, prolonged antibiotic therapy (up to 12 months) without surgery is sufficient. The duration of treatment remains a matter of debate as reliable evidence to support decision is lacking. 5 Most authors agree that a too-short course of antibiotics render a higher risk of relapse and that initial IV therapy over 4 to 6 weeks is mandatory. In thoracic actinomycosis shorter antibiotic regimes (< 3 months) were performed with equally high cure rates especially in patients receiving concomitant surgical treatment for control of the mean infection focus. 24 In our opinion, the treatment duration has to be individualized and discussed interdisciplinary. The main requirement to terminate antibiotics is a complete resolution of the infiltrates or persistent stabilization of residual lesions (scar) documented by CT scan. Additionally, periodic clinical examinations and blood chemistry are recommended to early identify side effects potentially associated with long-term antibiotic therapy.

Penicillin is the substance of choice; with penicillin V administered three to four times daily due to its kinetics, which can decrease the compliance with the treatment. So, amoxicillin is an alternative with two doses a day. For pregnant or penicillin-allergic patients, tetracycline and erythromycin provide an option. 25 Additionally, the dental status and oral hygiene of the patient should be checked and remediated if required. 26

Surgery for thoracic actinomycosis is used mostly for diagnostic purposes, especially to rule out malignancy or as a therapeutic adjunct in cases with complications as empyema, chest wall fistula, or hemoptysis. In our cases, surgical debridement of the abscess cavity without resection of underlying structures and a short course of vacuum wound dressing allowed a rapid decrease of the initial lesion and an excellent and durable closure of the chest wall fistula. Antibiotic therapy remains the cornerstone of successful treatment.

Empyema necessitatis due to unspecific empyema is a very rare entity leaving tuberculosis (mycobacteria), actinomycosis and malignancies as the most frequent differential diagnosis for fistulating diseases of the chest wall. Differentiation between these entities can be challenging. 27 We suggest actinomycosis should be kept in mind. Despite the causative organism does not play any role for initial surgical treatment gathering of samples at that point of time including tissue and liquid samples for diagnostics is crucial for the patient and the continuative therapy. With this strategy, an early diagnosis is possible and may provide the necessary information for the right therapy and may spare the patient from unnecessary isolation and extensive surgery.
